# Development of maternal and neonatal composite outcomes for trials evaluating timing of delivery in women with pre-eclampsia

**DOI:** 10.1186/1745-6215-14-S1-P50

**Published:** 2013-11-29

**Authors:** Fiona Fong, Ewelina Rogozinska, John Allotey, Steve Kempley, Divyen Shah, Shakila Thangaratinam

**Affiliations:** 1 Women's Health Research Unit, Barts and the London School of Medicine and Dentistry, London, UK; 2 Centre for Paediatrics, Blizard Institute, Barts and the London School of Medicine and Dentistry, London, UK; 3 Neonatal Unit, Barts Health NHS Trust, London, UK; 4 Multidisciplinary Evidence Synthesis Hub, Queen Mary University London, London, UK

## 

Composite outcomes comprising more than one clinically relevant end point are used in clinical trials where there is no single important outcome or the outcome is very rare. Pre-eclampsia is associated with significant maternal and neonatal complications and delivery is often expedited to minimise complications. There is a need for a randomised trial to evaluate the timing of delivery in women with mild to moderate pre-eclampsia at late preterm gestation. No single outcome has been identified to be the most clinically important, reflecting the multisystemic nature of pre-eclampsia. We developed composite maternal and neonatal outcomes to be considered as the primary outcome measure for a clinical trial in this area.

A two-generational Delphi method was used to identify these clinically important maternal and neonatal outcomes. Composite outcomes were developed based on biological plausibility, independence from each other, equal importance and frequency of occurrence. The final maternal composite outcome included maternal death, eclampsia, stroke or reversible ischaemic neurological deficit, pulmonary oedema, major obstetric haemorrhage, infusion of a third anti-hypertensive or need for positive ionotropic support, HELLP (haemolysis, elevated liver enzymes and low platelets) syndrome and placental abruption; and the neonatal composite outcome included neonatal death, respiratory distress syndrome needing ventilator support and neurological outcomes as cystic periventricular leukomalacia and grade III/IV intraventricular haemorrhage.

The composite outcomes developed will enable clinical trials on the timing of delivery in women with mild to moderate pre-eclampsia to provide robust estimates for the intervention to be implemented in clinical practice.

